# Improving mental health of adolescents with Type 1 diabetes: protocol for a randomized controlled trial of the *Nothing Ventured Nothing Gained* online adolescent and parenting support intervention

**DOI:** 10.1186/1471-2458-13-1185

**Published:** 2013-12-17

**Authors:** Naomi J Hackworth, Jan Matthews, Kylie Burke, Zvezdana Petrovic, Britt Klein, Elisabeth A Northam, Michael Kyrios, Lisa Chiechomski, Fergus J Cameron

**Affiliations:** 1 Parenting Research Centre, 5/232 Victoria Pde, 3002, East Melbourne, Victoria, Australia; 2 Parenting and Family Support Centre, School of Psychology, University of Queensland, Brisbane, Queensland, Australia; 3 University of Ballarat, Mt Helen, Victoria, Australia; 4 The Australian National University, Canberra, Australian Capital Territory, Australia; 5 Swinburne University of Technology, Hawthorn, Victoria, Australia; 6 Hormone Research, Murdoch Children’s Research Institute, Parkville, Victoria, Australia; 7 Department of Psychology, Royal Children’s Hospital, Parkville, Victoria, Australia; 8 School of Psychology, University of Melbourne, Melbourne, Victoria, Australia; 9 Department of Endocrinology and Diabetes, Royal Children’s Hospital, Parkville, Victoria, Australia; 10 Murdoch Children’s Research Institute, Parkville, Victoria, Australia; 11 Department of Pediatrics, University of Melbourne, Melbourne, Victoria, Australia

**Keywords:** Diabetes, Mental health, Parenting, Adolescents, Online intervention

## Abstract

**Background:**

Management of Type 1 diabetes comes with substantial personal and psychological demands particularly during adolescence, placing young people at significant risk for mental health problems. Supportive parenting can mitigate these risks, however the challenges associated with parenting a child with a chronic illness can interfere with a parent’s capacity to parent effectively. Interventions that provide support for *both* the adolescent and their parents are needed to prevent mental health problems in adolescents; to support positive parent-adolescent relationships; and to empower young people to better self-manage their illness. This paper presents the research protocol for a study evaluating the efficacy of the *Nothing Ventured Nothing Gained* online adolescent and parenting intervention which aims to improve the mental health outcomes of adolescents with Type 1 diabetes.

**Method/Design:**

A randomized controlled trial using repeated measures with two arms (intervention and wait-list control) will be used to evaluate the efficacy and acceptability of the online intervention. Approximately 120 adolescents with Type 1 diabetes, aged 13–18 years and one of their parents/guardians will be recruited from pediatric diabetes clinics across Victoria, Australia. Participants will be randomized to receive the intervention immediately or to wait 6 months before accessing the intervention. Adolescent, parent and family outcomes will be assessed via self-report questionnaires at three time points (baseline, 6 weeks and 6 months). The primary outcome is improved adolescent mental health (depression and anxiety). Secondary outcomes include adolescent behavioral (diabetes self-management and risk taking behavior), psychosocial (diabetes relevant quality of life, parent reported child well-being, self-efficacy, resilience, and perceived illness benefits and burdens); metabolic (HbA1c) outcomes; parent psychosocial outcomes (negative affect and fatigue, self-efficacy, and parent experience of child illness); and family outcomes (parent and adolescent reported parent-adolescent communication, responsibility for diabetes care, diabetes related conflict). Process variables including recruitment, retention, intervention completion and intervention satisfaction will also be assessed.

**Discussion:**

The results of this study will provide valuable information about the efficacy, acceptability and therefore the viability of delivering online interventions to families affected by chronic illnesses such as Type 1 diabetes.

**Trial registration:**

Australian New Zealand clinical trials registry (ANZCTR); ACTRN12610000170022

## Background

Type 1 diabetes (T1D) is a serious, life-long chronic illness most often emerging before or during adolescence. With a population prevalence between 136 (USA) and 428 (Finland) of 100,000 children and increasing global incidence, T1D is one of the most significant chronic illnesses emerging in childhood [1]. Treatment of T1D is intensive and ongoing, requiring a strict daily regimen of insulin injections and monitoring of blood glucose levels and dietary intake to avoid episodes of extremely high or extremely low blood glucose, both of which can be life threatening [1]. T1D is associated with serious long-term health complications such as kidney and heart disease, circulatory problems, retinopathy, and neuropathy [2] and a range of co-morbid mental health disorders including depression, anxiety, conduct and eating disorders [3-6]. A number of large scale trials have shown that optimal metabolic control, achieved through intensive disease management, reduces the risk of long term health complications (e.g., the Diabetes Control and Complications Trial [7]). However, intensive diabetes self-management imposes significant personal and psychological demands and is difficult to sustain.

Diabetes self-management has been shown to be particularly poor during adolescence [4], a time when young people undergo significant social, emotional and physical changes. These changes can sometimes result in the development of mental health difficulties or the commencement of dangerous risk taking behaviors (e.g., alcohol and other drug use) [4-6,8].

### Mental health outcomes for adolescents with T1D

The inter-relationship between mental health and physical health outcomes in adolescents with T1D is complex. Co-morbid depression and anxiety in adolescents with T1D has been associated with poorer disease adjustment and metabolic control [9], and more frequent hospitalization [10]. Conversely better metabolic control has been found to be associated with higher levels of internalizing symptoms [11] and meticulous adherence to diabetes self-care has been linked to higher anxiety [12], suggesting there is a psychological burden in maintaining the strict treatment regimen required to achieve optimum control. Indeed, evidence emerging from a number of longitudinal studies suggests that adolescents with T1D are more than twice as likely to develop serious mental health outcomes (e.g., mood, anxiety, eating or behavior disorders) than their non-diabetic peers [5]. The health of adolescents with T1D can also be compromised by risk taking and other health compromising behaviors often apparent in adolescence (e.g., alcohol consumption, poor eating and sleep practices). It is therefore critical to better understand how to support young people with T1D to manage those risks and to engage in health promoting behaviors.

### Family interventions to support adolescents with T1D

Parents are one of the primary influences on their children and play a critical protective role in mediating the health and well-being of their adolescent children [13,14]. For example, parent-adolescent relationships that are warm, loving, and involve clear boundaries and effective monitoring are associated with the development of resilience and effective coping resources in the adolescent [15,16]. Among adolescents with T1D, supportive parenting is associated with improved diabetes management and quality of life [17-20]. In addition, examination of parent-adolescent transactional processes has revealed that increased parent distress is associated with poorer adjustment in adolescents with T1D (e.g., [21,22]).

Parenting an adolescent with T1D can be challenging in that parents must adapt their parenting to include attention to their child’s medical care, and many report heightened anxiety, worry and frustration about their parenting role [23,24]. Most parents display signs of stress and anxiety within a very short time of their child’s diagnosis [25] and typically report grief, loss and relationship stress as well as family and work disruption [26]. Thus, in addition to care and support for the adolescent with T1D, attention to parenting and the needs of parents is warranted.

Parenting programs have been shown to be effective in producing positive outcomes for both parents and their children across a diverse range of childhood behavioral and health difficulties [27-29]. Programs that provide parents with strategies to help them build and maintain supportive relationships with their adolescent and to deal with the difficult behaviors that arise during the adolescent years have been shown to be particularly effective [30,31].

While a number of reviews have demonstrated some benefits of family interventions in improving outcomes for children with T1D [12,32], such interventions have primarily focused on improving treatment adherence behaviors [12,33] rather than easing the psychological burden of the disease. Interventions for adolescents that focus on increasing coping and teaching problem solving skills have been found to be effective in addressing the psychological barriers to self-management when delivered across a range of modalities, although their impact on longer term adherence and metabolic control is yet to be established [34].

Interventions that target *both* the adolescent and their parent offer the potential to prevent mental health problems in the adolescent through affecting the context in which adolescent difficulties occur, as well as improving parent well-being [35-38].

### The service context

The current availability of intervention and support for families with a child with T1D is variable, as is the way in which families cope with the illness [26]. Little attention has been given to provision of support for parents that assists them to deal with the emotional impact of their child’s health condition or their own well-being [39]. Such a focus appears important given the documented links between parent well-being and child outcomes found in the child development, risk and protection literature [40,41]. Furthermore, not all families have easy access to health services and those experiencing geographical or social isolation or who are unable to access health services due to time, transport or other constraints are at increased risk due to limited or no contact with professional and other supports.

While research shows that individual and group face-to-face interventions have been effective, the internet is one potential avenue for improving the accessibility of interventions that may otherwise be difficult to access due to geographic and social isolation. There is growing evidence for the efficacy of online mental and physical health interventions targeting young people (see [42-44]) and for parents of young people with T1D [45] demonstrating the viability of an internet intervention for adolescents with T1D and their parents. An internet-based intervention is also likely to be more engaging for adolescents who are early adopters of new technology [46], and for parents and adolescents for whom access to conventional services is not possible due to geographical and/or social isolation (e.g., due to study or work commitments). Most people, including those from disadvantaged backgrounds, have access to the internet, with approximately 95% of 15 to 25 year olds, 96% of 35 to 49 year olds, and 84% of 50 to 64 year olds regularly using the internet in Australia [47,48] – similar rates to those observed in the United States [49]. Taken together these findings support the use of internet-based health programs for education about, and prevention and treatment of psychological distress, and enhancement of optimal self-management of chronic illness during adolescence.

### The current study

This paper presents the study protocol for the randomized controlled trial (RCT) of the *Nothing Ventured Nothing Gained* intervention (*NVNG*), an online adolescent and parenting intervention that aims to improve the physical and mental health outcomes of adolescents with T1D and their parents. To date, there is no published research on the efficacy of online interventions with both an adolescent *and* parenting component that aims to improve outcomes for adolescents with T1D.

### Primary outcomes

Primary outcomes will be adolescent mental health (depression and anxiety). Our primary hypothesis is that adolescents randomized to receive the *NVNG* intervention immediately, will demonstrate significantly better mental health (lower levels of depression and anxiety) than adolescents in the wait-list control group, from pre- to post-intervention and at the 6 month follow-up.

### Secondary outcomes

Secondary outcomes will be adolescent behavioral (diabetes self-management and risk taking behavior), psychosocial (diabetes relevant quality of life, parent reported child well-being, self-efficacy, resilience, and perceived illness benefits and burdens); metabolic (HbA1c) outcomes; parent psychosocial outcomes (negative affect, fatigue, self-efficacy and parent experience of child illness); as well as adolescent and parent reported family outcomes (parent-adolescent communication, responsibility for diabetes care, diabetes related conflict). Our secondary hypotheses are: a) Adolescents randomized to receive *NVNG* immediately, compared to those who are required to wait for 6 months, will demonstrate significantly greater improvements in well-being (diabetes quality of life, parent-reported well-being), cognitive (self-efficacy, resilience, perceived illness benefits and burdens), and behavioral (diabetes self-management, reduced risk taking) outcomes from pre- to post-intervention and at the 6 month follow-up; b) Parents randomized to receive *NVNG* immediately, compared to those who wait for 6 months, will demonstrate significantly better mental health (lower levels of depression, anxiety and stress), from pre- to post-intervention and at the 6 month follow-up; and c) Parents and adolescents randomized to receive *NVNG* immediately, will demonstrate significantly greater improvements in family communication, family conflict, and shared responsibility for diabetes management than that observed in the wait-list control group.

The results of this study will provide valuable information about the efficacy, acceptability and therefore the viability of delivering online intervention to families affected by chronic illness such as T1D.

## Methods/Design

The study will evaluate the efficacy, acceptability, and usability of the *NVNG* intervention with approximately 120 adolescents with T1D and their parents in Victoria, Australia. An RCT design, using repeated measures with two arms (intervention and wait-list control), will be used to assess the efficacy of the *NVNG* intervention. Outcomes for participants in both study arms will be assessed at three time points, at baseline (Time = 0), post-intervention/wait (Time = 6 weeks) and follow-up (Time = 6 months).

### Ethics approval

Ethics approval has been obtained from the Human Research Ethics Committees from the Royal Children’s Hospital (No. 29134) and Southern Health (No. 12103B).

### Participants

In Victoria Australia, the majority of adolescents with T1D receive their diabetes care from diabetes clinics within the state hospital system, by attending diabetes clinics approximately 3-monthly. This study will recruit adolescents and their parents from the two major pediatric diabetes clinics in Melbourne and their associated rural outreach clinics in Hamilton and Horsham, Victoria.

A rolling recruitment strategy will be used to recruit approximately 120 adolescents (60 intervention, 60 wait-list control group) with T1D, and one of their parents/guardians (n =120; 60 intervention; 60 wait-list control group). Researchers from the team will attend all pediatric diabetes clinic sessions over the course of four months for the purpose of face to face recruitment. Given that adolescents attend clinics 3-monthly, this will ensure that they reach all potential participants.

### Eligibility criteria

For adolescents, eligibility criteria will be a diagnosis with T1D, age between 13 to 18 years, access to the internet, and signed parental consent. For parents, eligibility criteria will include having a child aged between 13 and 18 years who has T1D, and access and ability to use the internet. Because the intervention and study questionnaires are available only in English language, adolescents and parents who are unable to understand spoken and written English will be excluded from participation.

### Sample size calculation

Given that there are no meta-analyses available for internet interventions of family centered parenting and adolescent programs, studies reporting face-to-face parenting and adolescent interventions and internet interventions directed at health behavior change were used to inform power calculations [50]. A meta-analysis of psychological interventions to improve outcomes in children and adolescents with T1D reported a moderate effect of family therapy on child/adolescent psychological distress (ES = .46) and HbA1c (ES = .41) [51]. A meta-analysis of internet interventions aiming to promote adolescent health related behaviors showed moderate effects (ES = .33) for interventions grounded in Cognitive Behavioral Theory or Theory of Planned Behavior [44]. Therefore a medium effect size is expected on the primary adolescent mental health outcome measure, as measured by the CDI and the RCMAS-S, between the two groups across the three assessment points. To power the study at the 80% level, based on an alpha level of .05, with an estimated medium effect size (.40) and with two groups over three assessment points, 64 complete responses will be required. Given that self-help online intervention studies often report high attrition rates (e.g. [52,53]), we have allowed for a 30% attrition rate between baseline and pre-assessment, and a further 20% attrition rate between post and follow-up; therefore a total of approximately 120 participants will be required; 60 per group.

### Recruitment

Participants will be recruited during routine visits to their diabetes clinic, where researchers will introduce the study in person to all families who meet the eligibility criteria. Those who express an interest will be given a Participant Information and Consent Form and a Registration Form to complete and return to the researchers in a reply paid envelope. Parents will be asked to nominate the primary and secondary carer in their family. Only the primary carer will participate in the study. Whilst the researchers acknowledge that it would be preferable for both parents to participate where possible, the limitations in terms of sample size, mean that if some participants had two parents and some only one, intervention dosage would be a potential confound. Participants will also be recruited via online methods including advertising on websites and in newsletters of peak Diabetes organizations. Potential participants who contact the researchers to express an interest and who meet the eligibility criteria will be sent the Participant Information and Consent Form and Registration Form by mail.

### Study arms

Participants will be randomized to one of two treatment arms: Intervention or wait-list control. Participants from both the intervention and control arms of the study will continue to receive usual care form their regular diabetes clinics throughout the study. Participants in the Intervention arm will receive immediate access to the *NVNG* intervention. Following randomization, participants in the wait-list control arm will be told that they are on a waiting list for 6 months after which time they will be given access to the intervention. During the waiting period they will be sent email or text message reminders to complete their post and follow up questionnaires but will have no other contact with the research team during that time.

### Randomization procedure

**Figure 1:**
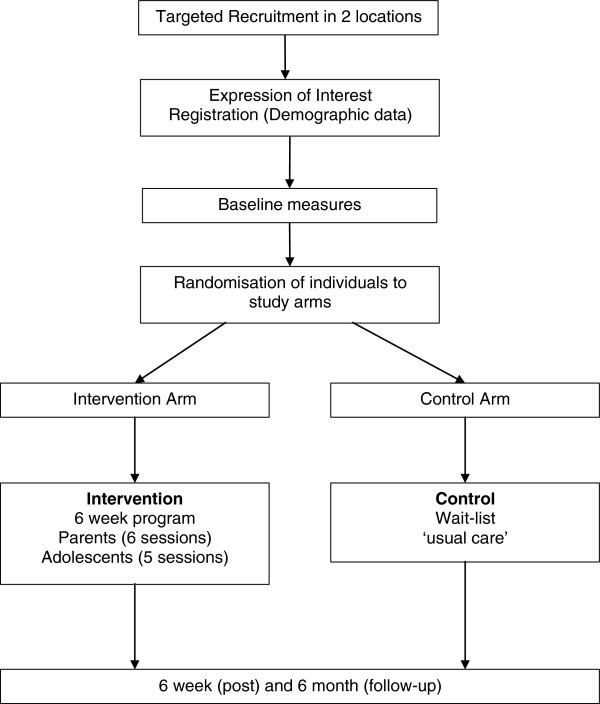
**CONSORT diagram for the randomized controlled trial of the *Nothing Ventured Nothing Gained intervention.***

In accordance with CONSORT guidelines [54,55], randomization will occur after participants have completed their baseline questionnaire. A Hypertext Pre-processor (PHP) randomization algorithm method will be used to randomize participants into one of the two study arms. Those who are randomized to the intervention arm will be given immediate access to the online intervention and those randomized to the control arm will be placed on the waiting list and given access to the intervention 6 months after completing the post questionnaire. The CONSORT diagram showing the flow of participants through the study is shown in Figure 1.

### *NVNG* online parent and adolescent intervention

*NVNG* incorporates two separate but parallel components: an adolescent intervention and a parent intervention. The intervention is designed for parents and adolescents to complete in parallel and is fully self-directed. Adolescents and parents will be provided with individual login details and will work through most sessions separately. However, some activities will be completed collaboratively (e.g., problem solving strategies). Participants will receive weekly email or text message reminders to log in to complete the sessions.

### Adolescent intervention

The adolescent intervention consists of five sessions completed over a six week period. The intervention is based on cognitive behavioral principles and incorporates a mixture of psycho-education and active learning exercises. The goal of the adolescent intervention is to increase their knowledge of the interface between their illness and adolescent development, to increase self-efficacy and coping, to provide strategies to improve mental and physical well-being, and to build and maintain positive relationships with family and friends.

### Parent intervention

The parent intervention consists of six sessions, intended to be completed over a six week period. The *NVNG* parent intervention aims to provide parents with information and skills to enable them to understand the interface between their children’s illness and normal adolescent development and to develop and maintain trusting, positive and accepting relationships with their adolescent thereby facilitating their transition to independent self-care within safe boundaries.

The intervention is based on the ABCD parenting young adolescents program [30], a group-based parenting program that combines a behavioral family intervention approach with acceptance-based strategies. ABCD targets adolescent behavior, parental well-being, and positive parenting practices. The program incorporates strategies designed to assist parents to maintain a close relationship with their adolescent as they move towards greater autonomy whilst still maintaining clear boundaries. Details of the adolescent and parent intervention content is shown in Table 1.

**Table 1:** Nothing ventured nothing gained Intervention overview

**Session theme**	**Content**
*Adolescent intervention*	
Session 1: Adolescent development and diabetes management	● Adolescence: physical, cognitive and social changes during adolescence.
	● Challenges of managing T1D during adolescence.
	● Links to T1D management resources.
Session 2: Connection between thoughts, feelings and behavior	● Understanding the relationship between thoughts, feelings and behaviors.
	● Recognizing signs of stress, anxiety and depression.
	● Recognizing and challenging unhelpful thoughts.
	● Accessing support.
Session 3: Communication	● Communicating with parents and handling difficult conversations.
	● Importance of body language, assertiveness, and confidence.
	● Communicating when feeling angry and frustrated.
Session 4: Building strong relationships	● Identifying values relating to relationships.
	● Relationships with parents and friends.
	● Steps to effective problem solving.
	● The effects of and managing bullying.
Session 5: Staying healthy	● Finding the balance between diet, exercise, work, sleep and leisure time.
	● Barriers to a healthy balance: peer pressure, smoking, alcohol, and other drugs.
	● Maintenance: Setting goals and problem solving to maintain the balance.
*Parent intervention*	
Session 1: Raising an adolescent with diabetes	● Adolescence: physical, cognitive and social changes during adolescence.
	● Adolescent cognitive processes and how they impact parent-adolescent interactions.
	● Challenges of managing T1D during adolescence.
	● Links to T1D management resources.
Session 2: Strengthening the parent-adolescent relationship	● Parenting values.
	● Ways of connecting with adolescents.
Session 3: Adolescent independence and safety	● A model for adolescent autonomy granting.
	● How to effectively use praise.
	● Demonstrating acceptance.
Session 4: Communication and problem solving	● Listening and talking to adolescents.
	● Barriers to communication.
	● Problem solving.
Session 5: Setting boundaries	● Limit setting and rules.
	● Characteristics of effective consequences.
Session 6: Parent self-care	● Parent health and well-being.
	● Recognizing and accessing support.
	● Managing stress.

### Measures

Outcomes will be assessed via self-administered adolescent and parent-report questionnaires. Measures were chosen for their sound psychometric properties, and their relevance to the primary and secondary outcomes. Participants may complete assessments online or via a pen and paper version (according to participant preference).

### Glycosylated hemoglobin (HbA1c)

Metabolic outcomes will be measured using glycosylated hemoglobin (HbA1c) which is a measure of glycemic control based on average blood glucose concentration levels in the 3 to 4 month period prior to the HbA1c test [56,57]. HbA1c levels which are collected as part of the adolescents’ routine care at the regular clinic visit will be obtained from the participants’ endocrinologist at four time points over a 12 month period (two levels prior to commencement in the study, and the two levels following completion of the intervention/waiting period).

Parent and adolescent self-report instruments used to collect data from participants, including primary and secondary outcomes, are listed in Table 2.

**Table 2:** Parent and adolescent self-report measurement tools and questions at each time point

		**Time point**		
	**Measurement tools/questions**	**Baseline**	**Post**	**Follow-up**
**Demographics**				
Adolescent characteristics	Age, gender, postcode, family Characteristics, living arrangements, education, age at diagnosis, family history of diabetes, diabetes treatment regime, number of hospitalizations, general medical history (Other Acute or chronic health conditions)	✓		
Parent characteristics	Age, gender, postcode, education, marital status, and employment			
**Process evaluation**				
Satisfaction with intervention**^**	6 item assessment rating the intervention with 4 additional open-ended questions regarding most liked, least liked, suggestions on improvements, and other comments		✓	
**Primary outcome**				
**Adolescent**				
Mental health	Children's depression inventory -short form (CDI -S) [58]	✓	✓	✓
	Revised children's manifest anxiety Scale (RCMAS -S) [59]	✓	✓	✓
**Secondary outcomes**		✓	✓	✓
**Adolescent**				
Behavioral	Self-care inventory (SCI-R) [60]	✓	✓	✓
	Adolescent risk taking questionnaire (ASQ) [61]	✓	✓	✓
Psychosocial	Diabetes quality of life survey for youths (DQoLY-S) [62]	✓	✓	✓
	Child health questionnaire: parent form 50 (CHQ-PF50) [63]	✓	✓	✓
	Stanford diabetes self-efficacy scale (SDSES) [64]	✓	✓	✓
	Benefit burden scale for children (BBSC) [65]	✓	✓	✓
	Adolescent resilience scale (ARS) [66]	✓	✓	✓
Metabolic	HbA1c	✓		✓
**Parent**				
Psychosocial	Depression, anxiety & stress scale (DASS) [67]	✓	✓	✓
	Fatigue assessment scale (FAS) [68]	✓	✓	✓
	Parent sense of competence scale (PSoC) [69]	✓	✓	✓
	Parent experience of child illness (PECI) [70]	✓	✓	✓
**Family**				
Communication	Family problem solving communication index (FPSCI)* [71]	✓	✓	✓
	Family management measure (FaMM) [72]	✓	✓	✓
Reduced conflict	Diabetes family conflict scale (DFCS)* [73]	✓	✓	✓
Diabetes responsibility	Diabetes family responsibility questionnaire (DFRQ)* [74]	✓	✓	✓
**Mediators and moderators**				
Perceived social support	Adolescent satisfaction with support from parents, boyfriend/girlfriend, friends, teachers, employers, diabetes specialists	✓	✓	✓
Other activities	Engagement in employment and extra-curricular activities.	✓		

^Administered to intervention group only.

*Administered to parents and adolescents.

### Process measures

Process variables, assessed at post-test include intervention dosage (% of modules accessed as recorded on the web interface), parent and adolescent perceptions of strengths and weaknesses of the intervention including appropriateness of the content, usability, satisfaction and acceptability of the web interface, and reason for non-participation/non-completion of the intervention (where relevant).

### Data analysis

Similarity of baseline characteristics of intervention and control participants will be assessed using appropriate summary statistics. Multi-level modeling will be used to compare the outcomes of participation in the intervention and wait-list control arms of the study in terms of pre-/post changes in primary and secondary outcomes while controlling for geographic region, age, gender, and other relevant confounds. Any imbalances in baseline characteristics between the study arms will also be controlled for in the model. Baseline data will be investigated to see if there are any significant factors associated with those participants who drop out in comparison to those that do not, to check for bias. By doing so we will be able to analyze the final results for attrition bias using the predicted probability of attrition as recommended by Heckman [75] and Rubin [76]. An intention to treat analysis and then a longitudinal hierarchical linear model analysis will be conducted, avoiding the need to impute missing data.

### Trial status

The trial is currently in the data collection phase. Recruitment to the study commenced in December 2011. A total of 231 parents and 236 adolescents (217 dyads, 14 individual parents and 19 individual adolescents) have volunteered to participate in the study. Of those parents and adolescents who volunteered to take part, 96 dyads, 38 individual parents and 23 individual adolescents completed self-report baseline measures (i.e. 134 parents and 119 adolescents). It is anticipated that full post and follow up data will be finalized in December 2013.

## Discussion

This paper provides a comprehensive description of the methods used to implement and evaluate the *NVNG* online parenting and adolescent intervention to prevent negative mental health outcomes in adolescents with T1D. As it is difficult to engage adolescents in their own treatment and self-care, the current research aims to trial an innovative online family centered approach (which to our knowledge is the first of its kind) that engages adolescents, in partnership with their parents, in mental health prevention and health promotion activities that are delivered via a medium that adolescents are typically keen to embrace – the internet. Because the intervention will be evidence-based and targets variables that have been shown to influence self-care behaviors (i.e. depression, anxiety, self-efficacy), it has the potential to have high impact on both physical and psychological outcomes for young people with Type1 diabetes. Furthermore, with online delivery, this intervention can be made available to adolescents and parents across a wide geographic area (national and international), many of whom may be disadvantaged by geographic isolation and a range of psychosocial obstacles. Once evaluated, interventions such as the one proposed in this study might potentially be a valuable resource in primary care settings to assist health professionals such as general practitioners, diabetes educators and community health workers to facilitate pathways to psychological care for their young clients.

## Competing interests

The authors declare that they have no competing interests.

## Authors’ contributions

NH drafted this paper and was responsible for research design and methodology and intervention development. JM, KB, BK and LC edited this paper and were involved in the conceptualization of the study, research design and methodology and intervention development. FC, EN and MK edited this paper, were involved in the conceptualization of the study, research design and recruitment methodology. ZP assisted with drafting this paper, and was involved in intervention development and study recruitment. All authors read and approved the final draft.

## Pre-publication history

The pre-publication history for this paper can be accessed here:

http://www.biomedcentral.com/1471-2458/13/1185/prepub
